# The loss in expectation of life after colon cancer: a population-based study

**DOI:** 10.1186/s12885-015-1427-2

**Published:** 2015-05-17

**Authors:** Therese M-L Andersson, Paul W. Dickman, Sandra Eloranta, Annika Sjövall, Mats Lambe, Paul C. Lambert

**Affiliations:** 1 Department of Medical Epidemiology and Biostatistics, Karolinska Institutet, Box 281, SE-171 77 Stockholm, Sweden; 2 Department of Molecular Medicine and Surgery, Karolinska Institutet, Center of Surgical Gastroenterology, Karolinska University Hospital, Stockholm, Sweden; 3 Regional Cancer Centre, Uppsala University Hospital, Uppsala, Sweden; 4 Department of Health Sciences, University of Leicester, Leicester, UK

**Keywords:** Colon cancer, Survival, Life expectancy, Population-based, Flexible parametric model, Life years lost, Sweden

## Abstract

**Background:**

To demonstrate how assessment of life expectancy and loss in expectation of life can be used to address a wide range of research questions of public health interest pertaining to the prognosis of cancer patients.

**Methods:**

We identified 135,092 cases of colon adenocarcinoma diagnosed during 1961–2011 from the population-based Swedish Cancer Register. Flexible parametric survival models for relative survival were used to estimate the life expectancy and the loss in expectation of life.

**Results:**

The loss in expectation of life for males aged 55 at diagnosis was 13.5 years (95 % CI 13.2–13.8) in 1965 and 12.8 (12.4–13.3) in 2005. For males aged 85 the corresponding figures were 3.21 (3.15–3.28) and 2.10 (2.04–2.17). The pattern was similar for females, but slightly greater loss in expectation of life. The loss in expectation of life is reduced given survival up to a certain time point post diagnosis. Among patients diagnosed in 2011, 945 life years could potentially be saved if the colon cancer survival among males could be brought to the same level as for females.

**Conclusion:**

Assessment of loss in expectation of life facilitates the understanding of the impact of cancer, both on individual and population level. Clear improvements in survival among colon cancer patients have led to a gain in life expectancy, partly due to a general increase in survival from all causes.

**Electronic supplementary material:**

The online version of this article (doi:10.1186/s12885-015-1427-2) contains supplementary material, which is available to authorized users.

## Background

The most commonly reported measure of cancer patient survival in population-based cancer studies is the 5-year relative survival ratio (RSR) [1]. It is a useful measure when comparing cancer survival over time or between groups as it should not be affected by varying mortality due to other causes. However, it is not easy to grasp what the RSR means in terms of the life expectancy of the patients. For example, increasing relative survival suggests that cancer care has changed for the better over time, although it does not necessarily lead to a decreasing loss in expectation of life. The loss in expectation of life of the patients, measured as the difference between the expected remaining life in the absence of cancer and the expected remaining life in the presence of cancer [2], also depends on temporal changes in the overall life expectancy. Therefore, investigating the impact of changes in survival in terms of loss in expectation of life should provide additional insight into studies of cancer patient survival and is potentially of greater interest for patients and clinicians. Moreover, the loss in expectation of life is also a measure of public health interest since it provides a better understanding of the impact of cancer in the population.

The loss in expectation of life can be quantified both at the individual and population level. For example, how many life years does a person of a particular age on average lose due to their cancer diagnosis (individual level), and what is the total number of life-years lost in a particular population (population level)? However, these measures are used very little in practice since estimation generally requires extrapolation of survival as the studies typically don’t follow all patients to the end of life. In a recent study, we showed that the loss in expectation of life can be reliably estimated using flexible parametric relative survival models [3], and we developed software to enable the estimation [4].

For the purpose of the present study, we demonstrate how estimation of loss in expectation of life can be used to address a wide range of research questions of public health interest pertaining to the survival and prognosis of colon cancer patients. Using data from the population based Swedish Cancer Register, the aim of the present study was three-fold. Firstly, to examine how life expectancy and loss of expectation of life for patients with colon cancer has changed over calendar time and to estimate the loss in expectation of life for recently diagnosed patients. Secondly, to estimate loss of expectation of life conditional on survival to, for example, one and five year after diagnosis, and thirdly to examine how many life-years can potentially be saved at a population level if sex differences in colon cancer survival could be eliminated.

## Methods

### Data

All incident cases of colon adenocarcinoma were identified in the nationwide population-based Swedish Cancer Register during the years 1961–2011. The Swedish Cancer Register was established in 1958. Clinicians and pathologists are independently required by law to notify the register about all new cases of cancer, which contributes to the completeness of the Swedish Cancer Register [5]. For the purpose of the present study, individuals with multiple records of primary colon adenocarcinomas were only included with their first recorded diagnosis. We excluded diagnoses that were detected incidentally at autopsy, individuals aged less than 20 at diagnosis or if the date of diagnosis was recorded to be after the date of death. All patients were followed-up until death, first emigration after diagnosis, 31/12-2012 or 15 years after diagnosis, whatever came first.

### Introduction to the statistical methods and concepts

Relative survival is defined as the ratio of the observed all-cause survival among cancer patients and the expected survival in a comparable group in the general population. It has become the preferred measure of cancer patient survival in population-based studies as it captures mortality that is either directly or indirectly related to the cancer without requiring information on cause of death [1]. The advantage of relative survival over cause-specific survival is that cause of death is not always available or reliable. Even if accurate information on cause of death is available it is often difficult to determine whether or not a death is due to the diagnosed cancer or not. For example it may not be obvious how to classify deaths that are secondary effects of treatment. The limitation of relative survival is that a comparable group in the general population has to be defined to obtain the expected survival. Population life tables are usually used, stratified on age, calendar year and sex, and the cancer patients are assumed to have the same expected survival as the general population. This assumption is generally feasible for colon cancer, but not for smoking-related cancers such as lung cancer where the patients would have a lower expected survival than the general population. It is interpreted as the proportion of patients still alive in the hypothetical scenario where cancer is the only possible cause of death. The mortality analogue to relative survival is excess mortality, defined as the difference between the observed all-cause mortality rate among cancer patients and the expected mortality rate in a comparable group in the general population.

The expectation of life from the date of cancer diagnosis until death (due to any cause) gives an estimate of the average number of years cancer patients are expected to live after they are diagnosed with cancer. The loss in expectation of life due to cancer is the difference between the anticipated expectation of life (in the absence of cancer), and the expectation of life among the cancer patients [2]. The anticipated expectation of life can be estimated from population mortality tables. Estimation of life expectancy generally requires extrapolation of the survival function, due to limited follow-up, since it requires all subjects to have died. It has been shown that the extrapolation of the observed survival function can be done reliably using flexible parametric survival models within a relative survival approach [3]. Flexible parametric survival models are fitted on the log cumulative hazard scale [4, 6, 7] and model the baseline hazard directly via restricted cubic splines and thus obviate the need to pre-specify a parametric distribution for the survival function. In this framework, various assumptions about the future excess mortality can be made, but since the long-term excess mortality constitutes a relatively small part of the all-cause mortality from around 6 or 7 years after diagnosis, the extrapolation is not heavily dependent on these assumptions.

Traditionally, cancer patient survival is estimated from time of diagnosis. However, from a clinical perspective it is also important to know how the survival changes as patients have survived several years after diagnosis. This is estimated by conditional survival probabilities. Similarly, the loss in expectation of life can be estimated conditional on surviving past a certain point after diagnosis, by estimating life expectancy and loss in expectation of life based on extrapolated conditional survival functions.

In studies where the purpose of the investigation is to provide estimates of the survival experience for recently diagnosed patients, a period approach to estimation (as opposed to a cohort approach) has been suggested and proven empirically superior [8, 9]. In a period analysis, only recently diagnosed patients contribute to the estimates of short term survival whereas patients diagnosed further in the past still contribute to estimates of long term survival. This set-up is made possible by pre-specifying a period window, and only person-time experienced within the period window contributes to the analysis.

### Modeling and estimation

To examine temporal trends in life expectancy and loss of expectation of life for patients with colon cancer a flexible parametric survival model for relative survival was fitted. The fitted model was used to extrapolate excess mortality beyond follow-up to enable estimation of life expectancy. Age at diagnosis, sex and year of diagnosis were included as covariates, with two-way interactions between all covariates and time-dependent effects. Age and year of diagnosis were modeled continuously and non-linearly using restricted cubic splines, and the results are presented for selected ages and calendar years.

To obtain estimates for recently diagnosed patients, from whom there is limited follow-up information, a period analysis was carried out with the period window set to 1/1-2007–31/12-2012, and a flexible parametric model was again fitted including the effects of age and sex.

Expected mortality rates, by age, sex and calendar year, were available up until 2011 and extrapolations of expected survival were based on population mortality projections from Statistics Sweden [10], estimated using the Lee-Carter method [11].

In order to quantify the impact of sex differences in survival, we applied the female cancer mortality rates to males (but keeping the male background mortality rates) to estimate what the loss in expectation of life for males would be if males had the same cancer patient mortality as females. This measure was used to calculate the total number of life years that would potentially be gained in the Swedish population if colon cancer survival among males could be brought to the same level as for females. In these calculations the total amount of life years lost for all patients in 2011 were contrasted to the corresponding total number of life years lost if males were given the female cancer mortality rates (as predicted from the model using period analysis).

All analyses were performed using Stata 12 (Statacorp, College Station, TX, USA). An extended description of the modelling assumptions and estimation is provided in Additional file 1. The study was approved by the Institutional Review Board at Karolinska Institutet.

## Results

### Descriptive statistics

Descriptive statistics for the 135,092 patients included in the study are presented in Table 1. The most common age group at diagnosis was 70–79 years (36 % of the patients). There were more females than males (52 vs. 48 %) and the annual number of patients diagnosed increased with calendar time. The total follow-up time was 635,449 person-years and 100,208 patients died during follow-up.

**Table 1:** Descriptive statistics for colon cancer patients diagnosed in Sweden during 1961–2011. *N* = number of diagnoses, *d* = number of deaths during follow-up^a^, % *d* = percentage dying during follow-up^a^

	1961–1971			1972–1981			1982–1991			1992–2001			2002–2011			Total		
	N (%)	d	% d	N	D	% d	N	D	% d	N	D	% d	N	D	% d	N	d	% d
Age																		
<50	1498 (7.3)	947	63.2	1180 (5.2)	716	60.7	1162 (4.4)	639	55.0	1282 (4.3)	632	49.3	1476 (4.2)	509	34.5	6598 (4.9)	3443	52.2
50–59	2800 (13.7)	1929	68.9	2667 (11.7)	1793	67.2	2480 (9.3)	1535	61.9	2905 (9.7)	1604	55.2	3280 (9.2)	1219	37.2	14,132 (10.5)	8080	57.2
60–69	5451 (26.7)	4481	82.2	5897 (26.0)	4567	77.5	6477 (24.4)	4665	72.0	6234 (20.9)	4059	65.1	8083 (22.8)	3113	38.5	32,142 (23.8)	20,885	65.0
70–79	6939 (34.0)	6506	93.8	8271 (36.4)	7557	91.4	10,015 (37.7)	8885	88.7	11,043 (36.9)	9130	82.7	12,224 (34.5)	5971	48.9	48,492 (35.9)	38,049	78.5
80+	3731 (18.3)	3684	98.7	4697 (20.7)	4634	98.7	6439 (24.2)	6313	98.0	8440 (28.2)	8156	96.6	10,421 (29.4)	6964	66.8	33,728 (25.0)	29,751	88.2
Males	10,014 (49.0)	8819	88.1	10,803 (47.6)	9426	87.3	12,507 (47.1)	10,712	85.7	14,451 (48.3)	11,712	81.1	17,435 (48.8)	9058	52.0	65,210 (48.3)	49,727	76.3
Females	10,405 (51.0)	8728	83.9	11,909 (52.4)	9841	82.6	14,066 (52.9)	11,325	80.5	15,453 (51.7)	11,869	76.8	18,049 (51.2)	8718	48.3	69,882 (51.7)	50,481	72.2
Total	20,419 (15.1)	17,547	85.9	22,712 (16.8)	19,267	84.8	26,573 (19.7)	22,037	82.9	29,904 (22.1)	23,581	78.9	35,484 (26.3)	17,776	50.1	135,092 (100)	100,208	74.2

^a^Patients are followed until death, first emigration after diagnosis, 31/12-2012 or a maximum of 15 years after diagnosis

### Temporal trends in life expectancy and loss of expectation of life

Table 2 shows the estimated 5-year RSR, the loss in expectation of life in years and the proportion of expected life lost, for males and females of selected ages and calendar years. The 5-year RSR decreased with increasing age for earlier years (e.g. 40.6 % (95 % CI 39.3–41.9) for males in 1965 aged 55 and 24.8 % (23.2–26.5) for males aged 85), but is fairly constant over age in 2005 (e.g. 61.5 % (60.5–62.6) for a males aged 55 and 59.8 % (58.5–61.1), for males aged 85). There was a clear increase in 5-year RSR over calendar time, and females in general had a higher 5-year RSR than males for all ages and calendar years.

**Table 2:** 5-year relative survival (5y RS), loss in expectation of life (LEL) and proportion of expected life lost (PELL) for selected ages, together with 95 % confidence intervals, for colon cancer patients diagnosed in Sweden during 1961–2011

		Age 55		Age 65		Age 75		Age 85	
		Males	Females	Males	Females	Males	Females	Males	Females
1965	5y RS (%)	40.6	41.9	36.5	38.2	31.3	33.3	24.8	27.0
		(39.3; 41.9)	(40.7; 43.2)	(35.5; 37.8)	(37.2; 39.2)	(30.2; 32.5)	(32.2; 34.4)	(23.2; 26.5)	(25.4; 28.6)
	LEL (years)	13.5	16.1	8.85	10.8	5.31	6.16	2.95	3.21
		(13.2; 13.8)	(15.8; 16.5)	(8.70; 9.00)	(10.6; 11.0)	(5.22; 5.40)	(6.06; 6.27)	(2.89; 3.01)	(3.15; 3.28)
	PELL (%)	60.9	59.6	62.8	61.3	64.5	62.9	68.8	67.3
		(59.6; 62.3)	(58.2; 60.9)	(61.8; 63.9)	(60.2; 62.4)	(63.4; 65.5)	(61.8; 63.9)	(67.4; 70.2)	(65.9; 68.7)
1975	5y RS (%)	44.3	46.0	41.5	43.4	37.4	39.7	31.9	34.5
		(43.2; 45.4)	(44.9; 47.0)	(40.6; 42.3)	(42.6; 44.3)	(36.5; 38.4)	(38.8; 40.7)	(30.4; 33.3)	(33.1; 35.9)
	LEL (years)	13.2	15.5	8.59	10.6	4.87	6.07	2.70	3.17
		(12.9; 13.4)	(15.2; 15.9)	(8.46; 8.72)	(10.5; 10.8)	(4.80; 4.95)	(5.98; 6.17)	(2.64; 2.75)	(3.11; 3.23)
	PELL (%)	57.3	55.4	58.3	56.4	58.1	56.7	60.5	59.1
		(56.2; 58.5)	(54.2; 56.6)	(57.4; 59.1)	(55.5; 57.3)	(57.3; 59.0)	(55.8; 57.6)	(59.3; 61.7)	(57.9; 60.3)
1985	5y RS (%)	51.3	53.2	49.7	51.8	47.0	49.5	42.6	45.5
		(50.4; 52.3)	(52.3; 54.1)	(48.9; 50.5)	(51.1; 52.6)	(46.1; 47.8)	(48.7; 50.2)	(41.3; 44.0)	(44.4; 46.7)
	LEL (years)	12.9	14.4	7.99	9.56	4.45	5.54	2.23	2.70
		(12.7; 13.2)	(14.1; 14.8)	(7.86; 8.11)	(9.40; 9.72)	(4.38; 4.52)	(5.45; 5.63)	(2.18; 2.27)	(2.65; 2.76)
	PELL (%)	51.3	48.7	50.9	48.6	49.3	47.7	48.7	47.6
		(50.2; 52.4)	(47.6; 49.8)	(50.1; 51.7)	(47.8; 49.4)	(48.5; 50.1)	(47.0; 48.5)	(47.6; 49.7)	(46.6; 48.6)
1995	5y RS (%)	56.5	58.5	56.0	58.3	54.5	57.1	51.5	54.5
		(55.5; 57.5)	(57.6; 59.4)	(55.2; 56.7)	(57.5; 59.0)	(53.7; 55.3)	(56.3; 57.8)	(50.2; 52.7)	(53.4; 55.6)
	LEL (years)	13.0	13.7	8.23	9.24	4.20	5.07	1.97	2.40
		(12.7; 13.3)	(13.4; 14.1)	(8.09; 8.38)	(9.06; 9.42)	(4.12; 4.27)	(4.97; 5.16)	(1.92; 2.02)	(2.34; 2.46)
	PELL (%)	47.8	44.6	46.6	43.6	43.1	41.2	40.2	39.1
		(46.6; 48.9)	(43.5; 45.8)	(45.7; 47.4)	(42.8; 44.5)	(42.3; 43.9)	(40.5; 42.0)	(39.2; 41.2)	(38.2; 40.1)
2005	5y RS (%)	61.5	63.6	62.0	64.3	61.5	64.1	59.8	62.7
		(60.5; 62.6)	(62.6; 64.6)	(61.2; 62.8)	(63.5; 65.1)	(60.6; 62.4)	(63.3; 64.9)	(58.5; 61.1)	(61.6; 63.9)
	LEL (years)	12.8	13.0	8.14	8.59	4.27	4.82	1.76	2.10
		(12.4; 13.3)	(12.5; 13.5)	(7.91; 8.38)	(8.33; 8.86)	(4.16; 4.38)	(4.69; 4.96)	(1.70; 1.81)	(2.04; 2.17)
	PELL (%)	44.2	40.6	42.2	38.7	38.3	35.7	33.5	32.3
		(42.6; 45.9)	(38.9; 42.3)	(41.0; 43.4)	(37.5; 39.9)	(37.3; 39.2)	(34.7; 36.7)	(32.5; 34.6)	(31.3; 33.2)

**Fig. 1:**
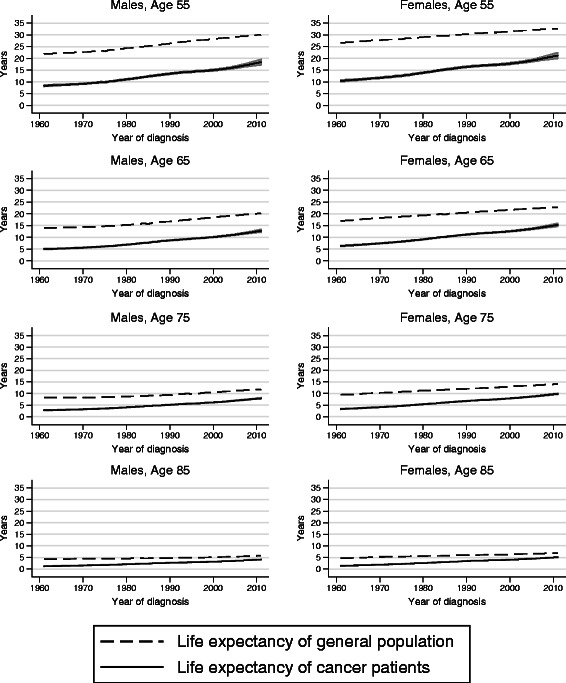
Temporal trends in life expectancy from diagnosis for colon cancer patients diagnosed in Sweden during 1961–2011

**Fig. 2:**
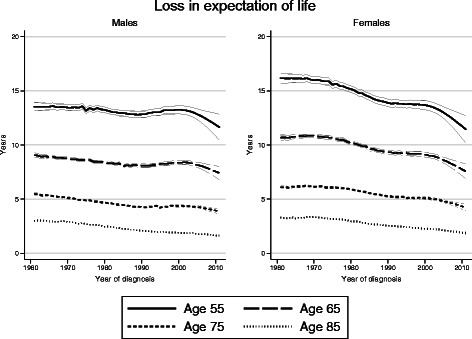
Temporal trends in the loss in expectation of life for colon cancer patients diagnosed in Sweden during 1961–2011

Figure 1 shows temporal trends in life expectancy from diagnosis for colon cancer patients and for a comparable disease-free general population. The difference between these two curves gives the loss in expectation of life. While the life expectancy for the colon cancer patients increased over calendar time, this increase mimics to a large extent the increase observed in the general population, and therefore the impact on the loss in expectation of life is modest (Table 2, Fig. 2). For example, for males aged 55 at diagnosis the loss in expectation of life was 13.5 years (95 % CI 13.2–13.8) in 1965 and 12.8 (12.4–13.3) in 2005. Female colon cancer patients have a better life expectancy than males, but since females in the general population have even higher life expectancy than males, the loss in expectation of life was greater among female patients.

There were pronounced age variations in life expectancy for cancer patients, with younger patients surviving longer. The loss in expectation of life decreased with age, since younger patients have a longer life expectancy in general. As an example, in 2005 males aged 85 lost on average 2.10 years, 95 % CI 2.04–2.17, compared to 12.8 years for males aged 55.

### Conditional loss in expectation of life

**Fig. 3:**
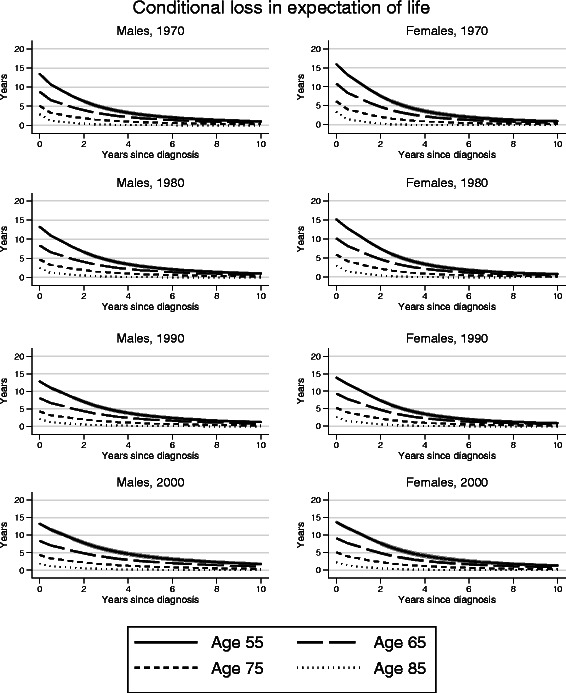
Loss in expectation of life conditional on time since diagnosis for colon cancer patients diagnosed in Sweden during 1961–2011

The loss in expectation of life decreased with follow-up time (Fig. 3), especially during the first few years post diagnosis. For female patients diagnosed in 2000 who had survived 5 years, the loss in expectation of life was 3.17 years (95 % CI 2.67–3.67) if diagnosed at age 55. For those diagnosed at age 65, 75 or 85 the corresponding conditional loss in expectation of life was 2.05 (1.80–2.29), 0.85 (0.74–0.97) and 0.06 (0.01–0.11) years respectively. After 8–10 years the life expectancy of the cancer patients was similar to that of the general population. The pattern was similar for males and across calendar years.

### Number of life years gained if males had the relative survival of females

The estimated total number of life years lost for the 3827 patients diagnosed in 2011 in Sweden was 23,480 years (Table 3). If males would have had the same cancer mortality as females (but still the background mortality of males) this would instead have been 22,535 years, giving a potential gain of 945 life years. Table 3 also shows the potential gain in loss in expectation of life at the individual level. Males aged 55 at diagnosis would on average live 0.93 years longer if they had the same cancer patient mortality as females aged 55 at diagnosis, whereas this number is 0.13 years for 85 year old males.

**Table 3:** Potential life years gained if sex differences in colon cancer patient survival in Sweden could be eliminated, for individual patients aged 55, 65, 75 and 85 and in the total cohort diagnosed in 2011

Age	Life years lost		Life years lost using female cancer mortality rates		Extra years of life using female cancer mortality rates	
	Males	Females	Males	Females	Males	Females
55	12.16	12.34	11.23	12.34	0.93	0
65	8.02	8.40	7.26	8.40	0.76	0
75	4.40	4.88	3.98	4.88	0.42	0
85	1.86	2.18	1.74	2.18	0.13	0
Population	11,843	11,637	10,898	11,637	945	0

## Discussion

Life expectancy and loss in expectation of life can be used to address a wide range of research questions of public health interest pertaining to the prognosis of cancer patients. We have demonstrated this by investigating life expectancy and loss in expectation of life after a diagnosis of colon cancer, how this changes over calendar time as well as from time of diagnosis, and by quantifying the survival difference between males and females.

Improved patient survival for cancer of the colon has been observed in Sweden and many other countries [12–16], reflecting improvements in adjuvant treatments as well as surgical techniques and peri-operative care that have led to an increasing proportion of patients safely operated on [17–19]. Of special note is that there seem to be only modest changes in the loss in expectation of life over time for colon cancer patients, since the improvements in the life expectancy in this patient group to a large extent have mimicked the improvement in life expectancy in the Swedish population. Whilst age is an important predictor of prognosis for many types of cancer, the 5-year RSR for colon cancer in Sweden is now very similar across age groups [16]. However, since young individuals have a longer life expectancy, a cancer diagnosis has a larger impact on loss in expectation of life. Thus, with regard to prognosis as a function of age, assessment of loss in expectation of life can provide a complement to e.g. the 5-year RSR since it gives more weight to young patients who have more years to lose.

Since cancer patient mortality changes with time since diagnosis, it is of interest to assess survival probabilities conditional on surviving up to a certain point [20, 21], and not only in survival measured from diagnosis. For that reason, it is of interest to present loss in expectation of life conditional on surviving up to a certain time point. This is of importance from the patient’s perspective, but also for health care planning. Our results show that the loss in expectation of life decreases substantially within the first few years after a colon cancer diagnosis and for those who had lived to 10 years post diagnosis, life expectancy was similar to that seen in the general population.

A small but persistent sex difference in survival of colon cancer patients has been observed in many studies, with males having a worse prognosis [12, 16, 22, 23]. Neither the reasons nor the impact of this difference is fully understood. It may reflect differences in sub site distribution [24–26], different anatomy with less efficient removal of lymph nodes at surgery among male patients [27], comorbidity burden [28] or differences in the likelihood of early diagnosis due to deep-rooted health care seeking behaviours [28, 29]. By estimating loss in expectation of life, we have shown that among patients diagnosed with colon cancer in 2011, 945 life years could potentially have been saved in Sweden if male patients had the same survival as female patients. Quantifying the impact of survival differences between groups in this way could also be used for exploring differences between socioeconomic groups, or between countries. This has been the focus of a large number of studies with some recent studies quantifying differences as the number of avoidable deaths [30–35]. A disadvantage of avoidable deaths is that the measure is highly time-dependent, since deaths can only be postponed and not avoided in the long run.

Another approach of comparing the life expectancy of cancer patients to the life expectancy of the general population is to estimate years of life lost (YLL) [36–38]. This is estimated by comparing the age at death of those dying due to cancer and the expected age at death for each individual or a pre-specified cut-off age (for example mean age at death in the population). One of the limitations with YLL is that its estimation relies on accurate cause of death classification. Another limitation is that this approach only includes patients that have died due to cancer, irrespective of when they were diagnosed, and therefore it cannot be used for a specific cohort of patients. Also, if a cut-off age is used, any differences between groups that occur after the cut-off age are ignored. The loss in expectation of life, on the other hand, is an informative measure for understanding the impact of cancer in the population, in a specific cohort and on the life expectancy for an individual.

Strengths of our study included the use of population-based data from the Swedish Cancer Register. The overall completeness of the Swedish Cancer Register has been estimated to 96 % [5]. Information on stage at diagnosis was not available in this material, since the Swedish Cancer Register did not collect stage information until 2003. Temporal trends in life expectancy and loss in expectation of life would certainly differ between stages. Survival for patients with stage I colon cancer has been high for decades, whereas survival for more advanced stages has improved over time [14, 39]. It could also be of interest to include information on treatment or local recurrence, but these data have only been reported nationally since 2007, in the Swedish Colorectal Cancer Registry. The only organized screening program in Sweden is in the Stockholm region [40] and cannot have had an impact on the estimated survival of the total study population. All inhabitants in Stockholm between 60–69 years old are invited to send in guaiac-based faecal occult blood tests. The screening program started in 2008, including two birth cohorts every year, so not all cohorts were included when the current study stopped. In total 165 cases of colorectal cancer were found through the organized screening between 2008 and 2011 [40] out of a total of 15,073 cases diagnosed between 2008 and 2011.

## Conclusion

In summary, assessing loss in expectation of life helps improve the understanding of the impact of a diagnosis of cancer and it is a good complement to the 5-year relative survival ratio. In this population-based study we have demonstrated how summarizing colon cancer survival in terms of loss in expectation of life can be useful in order to gain further insights of the impact of colon cancer on both the individual and population level by examining temporal trends, changes by time since diagnosis and quantifying differences between groups.

## Additional file

Additional file 1:
**Statistical modeling and estimation.**
