# Supplementary material for: The loss in expectation of life after colon cancer: a population-based study
Source: BMC Cancer. 2015 May 17;15:412. doi: 10.1186/s12885-015-1427-2 (PMC4493988; doi:10.1186/s12885-015-1427-2)
Supplement: Additional file 1: — Statistical modeling and estimation. [file 12885_2015_1427_MOESM1_ESM.docx]

**Additional file 1: Statistical modeling and estimation**

In a relative survival setting the overall survival among the cancer patients can be expressed as the expected survival, $S^{*}(t)$, multiplied with the relative survival (an estimate of the net survival associated with the disease),$R(t)$;

$S\left( t \right)=S^{*}\left( t \right)R(t)$.

Equivalently, the overall hazard of the cancer patients is the sum of the expected hazard, $h^{*}\left( t \right)$, and the excess hazard, $\lambda\left( t \right)$, associated with the cancer diagnosis;

$h\left( t \right)=h^{*}\left( t \right)+\lambda(t)$.

The expected survival, $S^{*}(t)$, and hazard, $h^{*}\left( t \right)$, are assumed to be known and obtained from population mortality rates, stratified by sex, age, year and possibly other covariates.

A flexible parametric survival model within a relative survival setting is fitted on the log cumulative excess hazard scale, using restricted cubic splines to estimate the baseline cumulative excess hazard;

$$\ln\left( \Lambda(t;\mathbf{z}) \right)=s\left( x,\boldsymbol{\gamma} \right)+\boldsymbol{z\beta}$$

where $\Lambda\left( t;\mathbf{z} \right)=\int_{0}^{t} \lambda(u;\boldsymbol{z})$ is the cumulative excess hazard, $s\left( x,\boldsymbol{\gamma} \right)$ is a restricted cubic spline function, $x=ln(t)$ and $\boldsymbol{z}$ are covariates. Time-dependent effects can easily be incorporated by including interactions between covariates and spline functions for log time. The relative survival function can be estimated from the model by the relationship between the survival and the cumulative hazard functions:

$$R\left( t;\boldsymbol{z} \right)=\exp\left( -\exp\left( \ln\left( \Lambda\left( t;\mathbf{z} \right) \right) \right) \right).$$

The loss in expectation of life is estimated as the difference between the expected and observed mean survival times;

$$\int_{0}^{t_{end}} S^{*}\left( t \right)dt-\int_{0}^{t_{end}} S^{*}\left( t \right)R\left( t;\mathbf{z} \right)dt,$$

where $S^{*}\left( t \right)$ is the expected survival function, and extrapolation beyond available follow-up can be done based on assumptions about the future population mortality rate. $R\left( t;\mathbf{z} \right)$ is the relative survival function estimated using a flexible parametric survival model and this can be extrapolated beyond available follow-up time using the spline parameters from the model. $t_{end}$ is the end point for extrapolation and should be chosen so that survival at that point is effectively zero. The integration is done numerically using Gaussian quadrature.

Loss in expectation of life conditional on surviving past year $x$ is estimated by integrating the conditional survival from time$x$,

$$\int_{x}^{t_{end}} {(S}^{*}(t))/(S^{*}(x))dt-\int_{x}^{t_{end}} {(S}^{*}(t)R\left( t;\mathbf{z} \right))/{(S}^{*}(x)R(x;\mathbf{z}))dt.$$

For the main analysis, sex, age at diagnosis and calendar year of diagnosis were included in the model. Age and year were modeled using restricted cubic splines with 4 degrees of freedom for the effect of age and 4 for the effect of year. Two-way interactions between the covariates were included, but only for the linear term of the splines for age and year. Time-dependent effects were also introduced for all main effects and interactions described. For the period analysis (with period window 1/1-2007 - 31/12-2012) age and sex were included in the model, age was again modelled continuously using restricted cubic splines with 4 degrees of freedom. An interaction between sex and the linear spline term for age was included, time-dependent effects were allowed for the main effects and the interaction. Both models had 5 degrees of freedom for the baseline and 3 degrees of freedom for the time-dependent effects.

In this study the expected mortality was estimated from population mortality rates stratified by age (given in years), sex and calendar year (obtained from www.mortality.org). Expected mortality rates were available up until 2011 and the same rates as in 2011were used for 2012. Extrapolations of expected survival beyond year 2012 and until 2070 were based on population mortality projections from Statistics Sweden, estimated using the Lee-Carter method. After 2070 the background mortality rates were assumed to be constant. The numerical integration was done using 40 nodes for the Gaussian quadrature rule, and was done up until 80 years after diagnosis to ensure that the survival function had reached zero even for the youngest ages.
